# Data supporting the optimization of liquid chromatography tandem mass spectrometry conditions to analyze EPA-priority hormones and bisphenol A in water samples

**DOI:** 10.1016/j.dib.2019.103958

**Published:** 2019-04-26

**Authors:** Ken Goeury, Sung Vo Duy, Gabriel Munoz, Michèle Prévost, Sébastien Sauvé

**Affiliations:** a Department of Chemistry, Université de Montréal, Montreal, QC, Canada; b Department of Civil, Geological and Mining Engineering, École Polytechnique de Montréal, Montreal, QC, Canada

## Abstract

This database presents the optimization of ultra-high-performance liquid chromatography electrospray ionization tandem mass spectrometry (UHPLC-MS/MS) for the analysis of EPA-priority endocrine disruptor compounds (13 hormones and bisphenol A). Various method parameters were tested and compared for improved sensitivity. Data related to the selection of the ionization source (heated-ESI *vs.* APCI) are presented, including optimization results of source parameters. Compound-dependent responses when varying the UHPLC mobile phase salt concentration of ammonium fluoride (NH_4_F) are supplied. Details on the chromatographic gradient program and chromatographic data demonstrating the separation of α-estradiol and β-estradiol are provided. In addition, we supply the details on mass spectrometry parameters under the optimized conditions, relative responses of quantification and confirmation MS/MS transitions (QT/CT), and number of points present in UHPLC-MS/MS spectra. The sample preparation and instrumental analysis procedures under the retained conditions are also described. The herein dataset supports the research “*Analysis of Environmental Protection Agency priority endocrine disruptor hormones and bisphenol A in tap, surface and wastewater by online concentration liquid chromatography tandem mass spectrometry*” Goeury et al., 2019.

**Table:** Specifications table

	
Subject area	*Analytical Chemistry*
More specific subject area	*Mass spectrometry and hormones/phthalates analysis*
Type of data	*Graphs, figures, tables, and chromatograms*
How data was acquired	*TSQ Quantiva triple quadrupole mass spectrometer from Thermo Scientific (Waltham, MA, U.S.A.), Xcalibur 3.0 software*
Data format	*Raw and analyzed output data*
Experimental factors	*Comparison of APCI and heated-ESI sources, MS parameters (sheath gas, auxiliary gas, sweep gas, spray voltage, collision energy, precursor ion, predominating transitions), acquisition mode (separate or fast polarity-switching), and mobile phase types (including NH*_*4*_*F concentration) for the detection of 13 hormones and bisphenol A at part-per-trillion levels*
Experimental features	*Robust online SPE – UHPLC-MS/MS method for the quantification of estrogens, progestogens, androgens and bisphenol A in water samples*
Data source location	*Montreal, Quebec, Canada*
Data accessibility	*Data is within this article*
Related research article	*K. Goeury, S. Vo Duy, G. Munoz, M. Prévost, S. Sauvé, Analysis of Environmental Protection Agency priority endocrine disruptor hormones and bisphenol A in tap, surface and wastewater by online concentration liquid chromatography tandem mass spectrometry, J. Chromatogr. A. (2019) 1–12.*https://doi.org/10.1016/j.chroma.2019.01.016*.*

**Table:** 

**Value of the data** • The data presented can be used by other scientists to monitor endocrine-disrupting compounds in water. • The data can be used to assist end-users with the selection of salt concentration, ionization source type, and mobile phases. • The data can also be used to implement chromatographic gradient conditions allowing the separation of α-estradiol and its β isomer. • The optimized instrumental parameters can be used in future LC-MS/MS method development and applications of hormones and bisphenol A.

## 1 Data

**Fig. 1:**
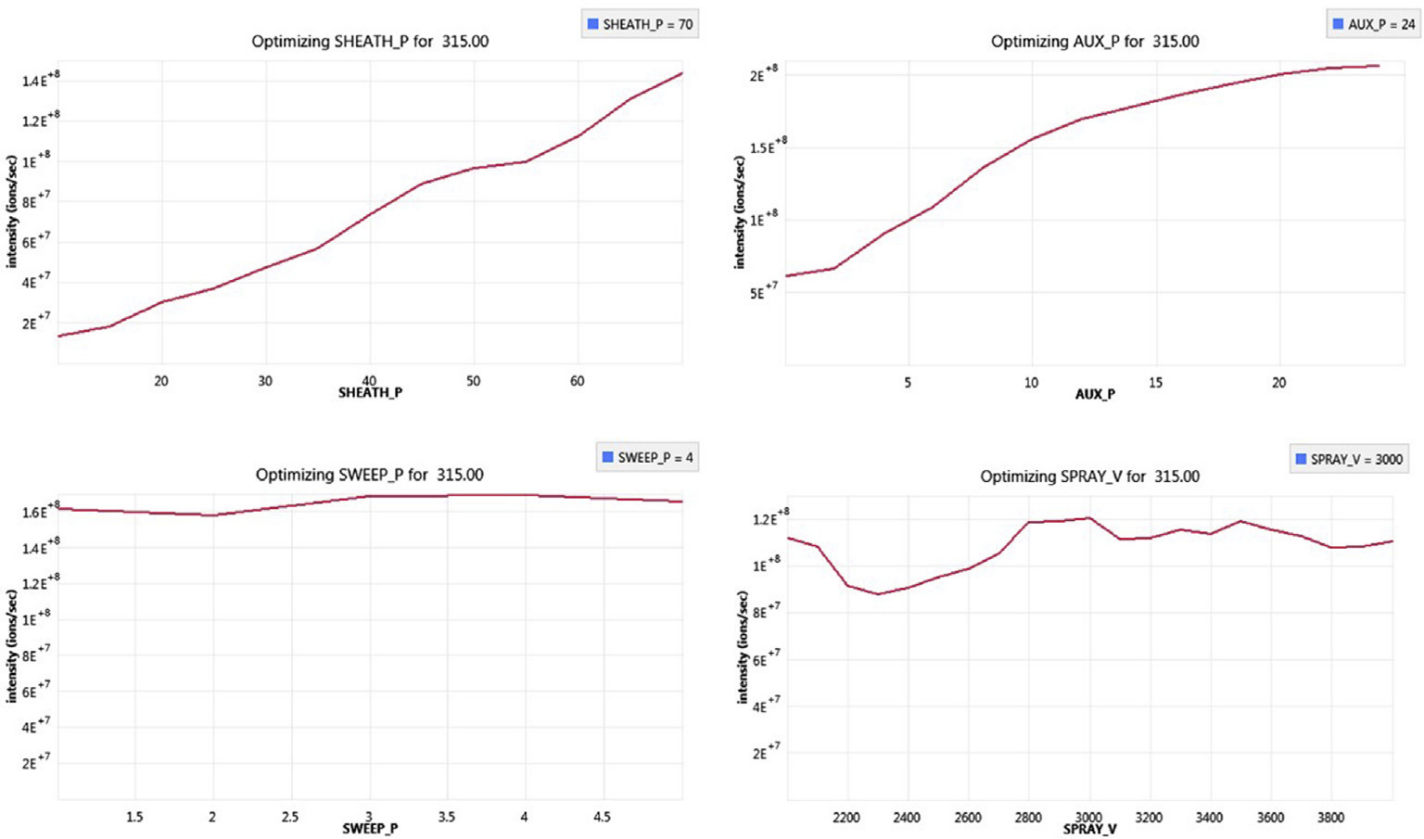
Acquisition reports for the optimization of sheath gas, auxiliary gas, sweep gas and spray voltage using the heated-ESI source with H_2_O/MeOH (50/50 v/v) as mobile phase for progesterone. The y-axis represents the intensity (counts/sec) while the x-axis represents either the gas flow in arbitrary units (Sheath_P, Aux_P, and Sweep_P) or the spray voltage (Spray_V).

**Fig. 2:**
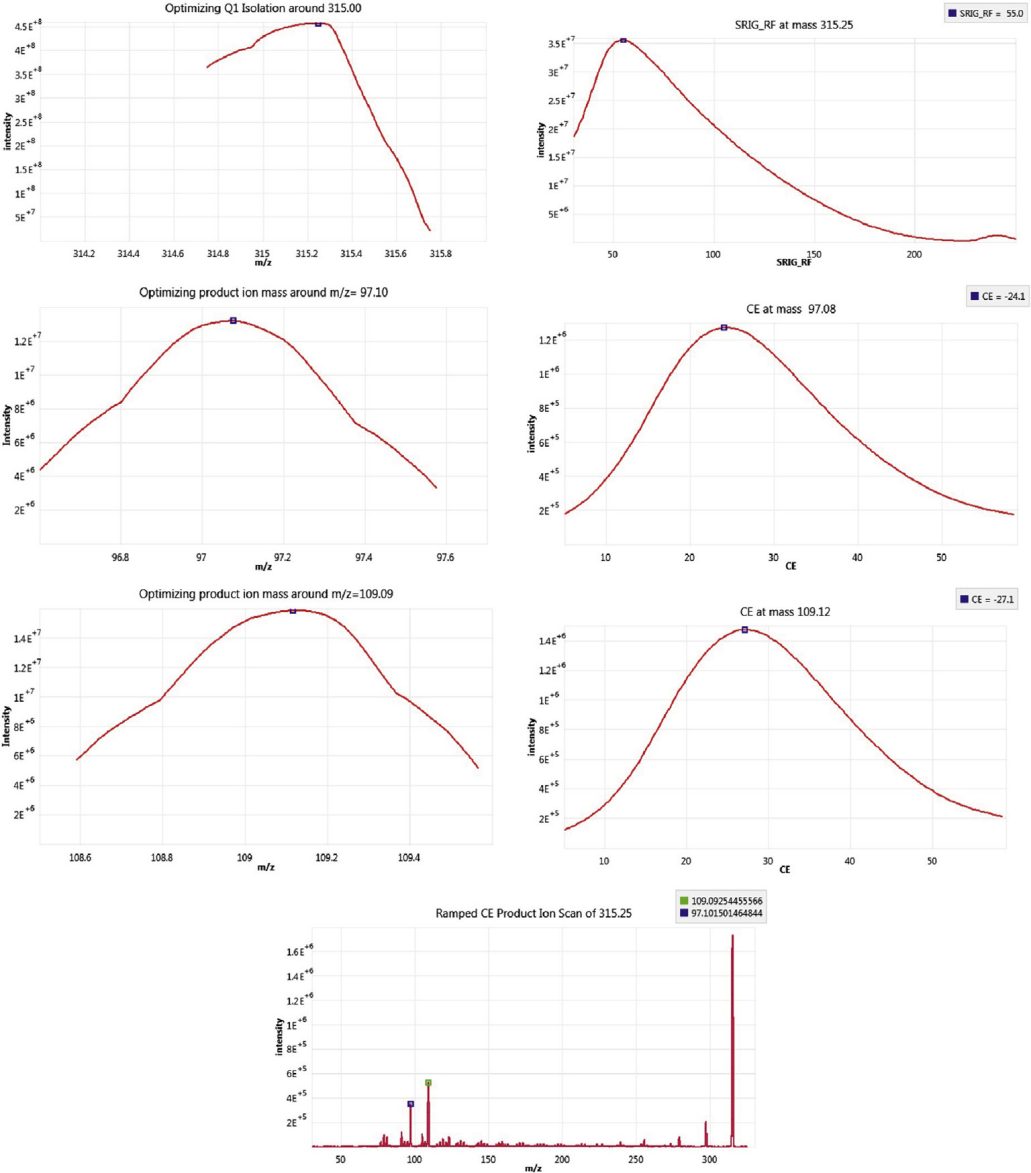
Acquisition reports for the optimization of precursor ion signal, fragment ion signal and the corresponding collision energy using the heated-ESI source with H_2_O/MeOH (50/50; v/v) as mobile phase for progesterone. The y-axis represents the intensity (counts/sec) while the x-axis represents either the mass-to-charge (m/z) ratio or the voltage for the collision energy (CE).

**Fig. 3:**
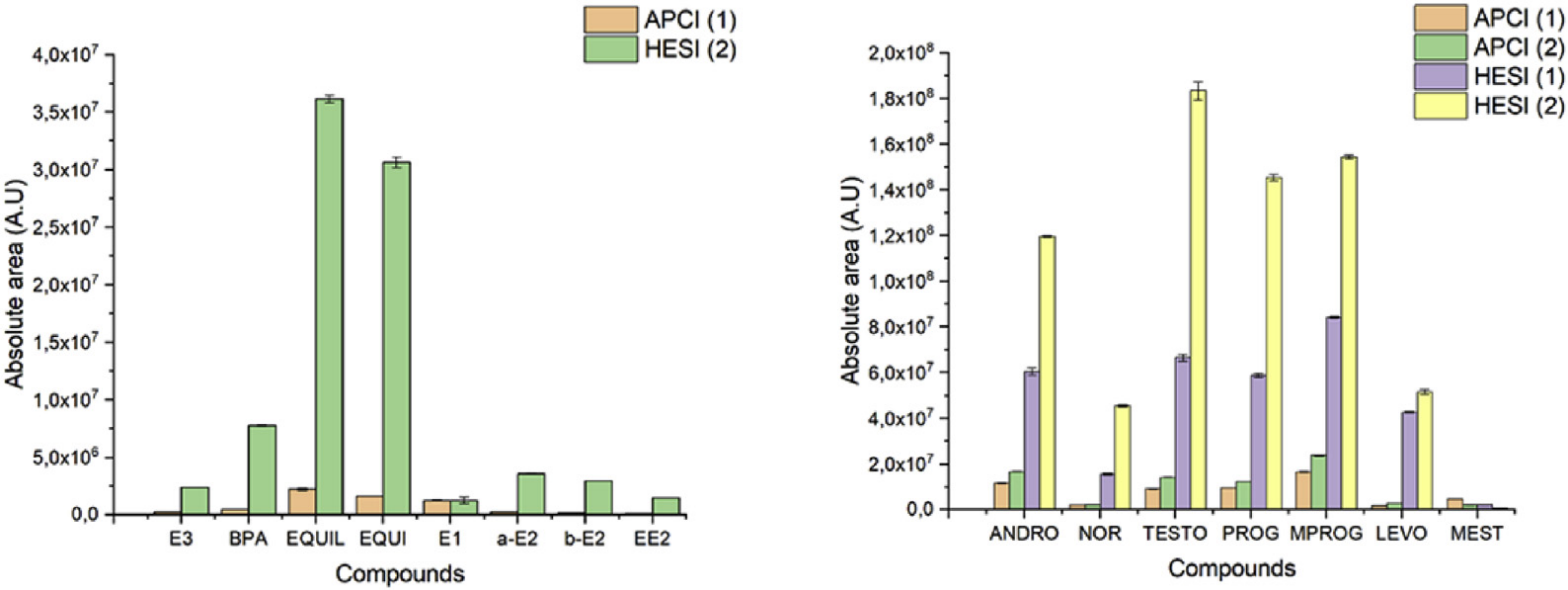
Absolute area of each compound analysed under different source types, APCI or heated-ESI (HESI), under the negative mode (left) and positive mode (right) acquisition. The tested mobile phases were as follows: (1) H_2_O+0.1% HCOOH/MeOH and (2) H_2_O/MeOH/NH_4_F(20mM). The absolute area is indicated in arbitrary units (A.U.).

**Fig. 4:**
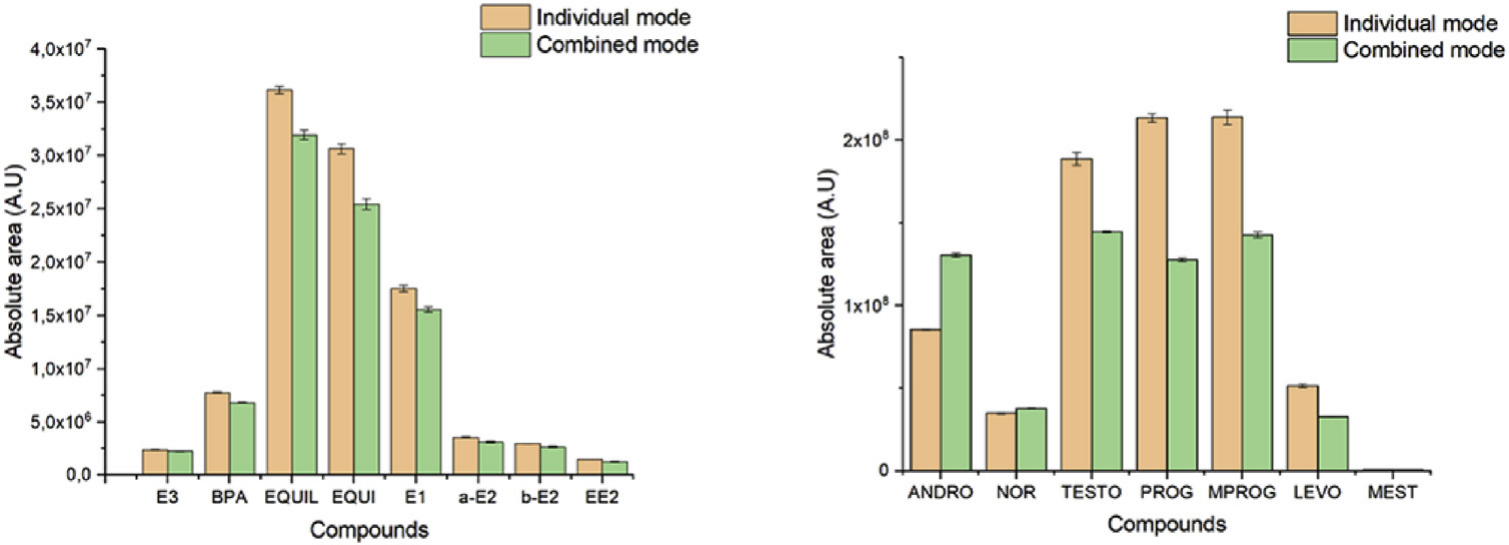
Absolute area of each compound analyzed under different mass spectrometry conditions, using the heated-ESI source for separate acquisition mode *vs.* combined positive/negative fast polarity-switching mode. Compounds are arranged according to their ionization (left panel: negative mode compounds; right panel: positive mode compounds). The absolute area is indicated in arbitrary units (A.U.).

**Fig. 5:**
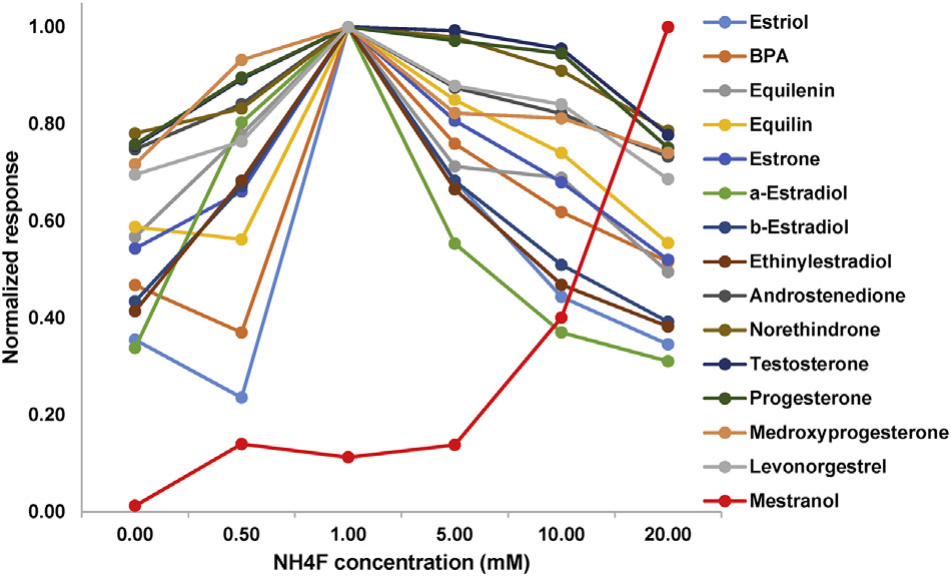
Normalized response of the targeted endocrine disruptor compounds, when the concentration of ammonium fluoride (NH_4_F) was varied in the range 0–20 mM (concentration in solvent C). For this test, we used the heated-ESI source in positive/negative polarity-switching mode.

**Fig. 6:**
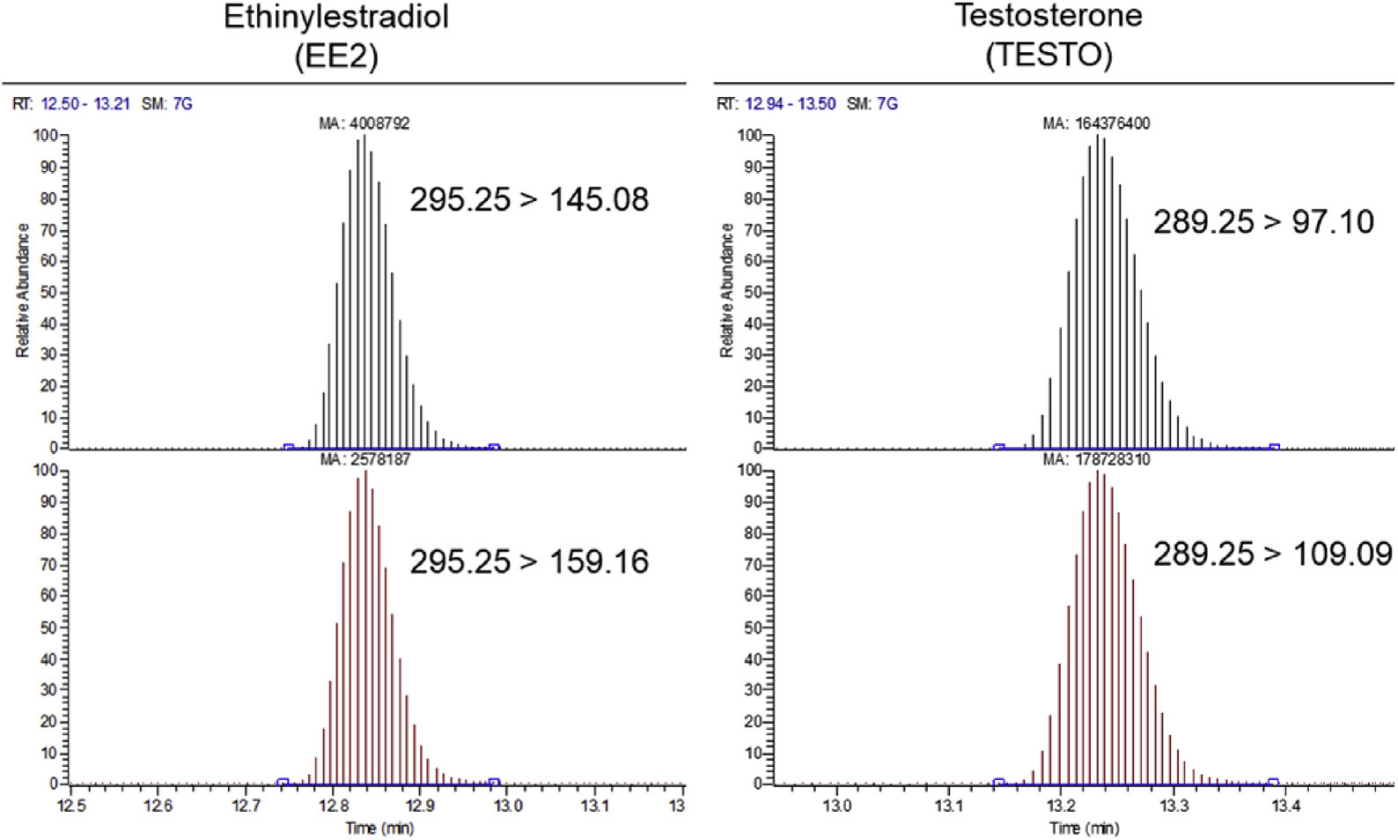
UHPLC-MS/MS chromatographic peaks in point by point view, illustrated for quantification and confirmation MS/MS transitions of ethinylestradiol (left) and testosterone (right). Each compound was verified to reach the U.S. EPA criterion that recommends a minimum of 10 points per peak [3].

**Fig. 7:**
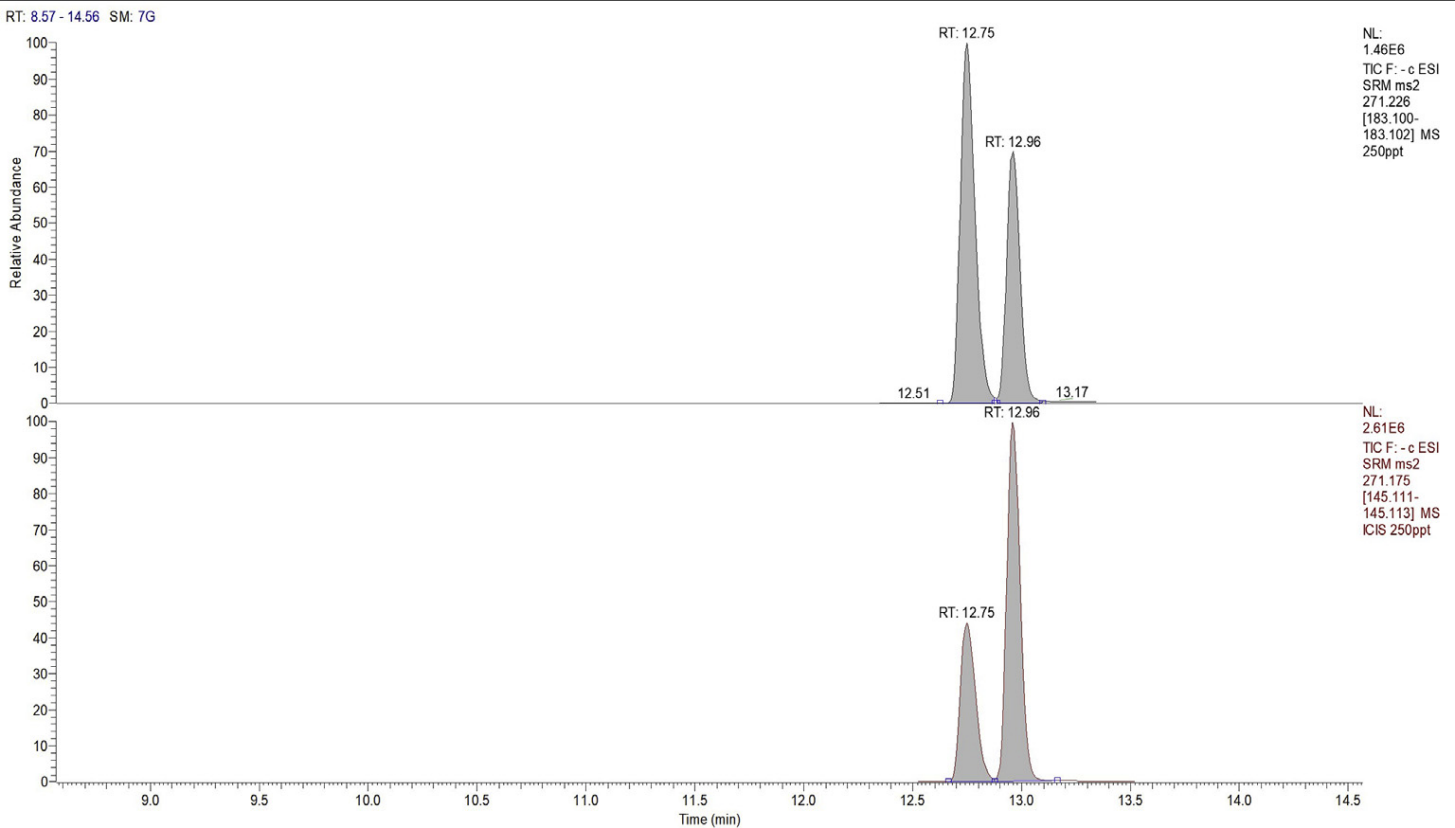
UHPLC-MS/MS chromatograms illustrating the separation of α-estradiol and β-estradiol isomers.

**Fig. 8:**
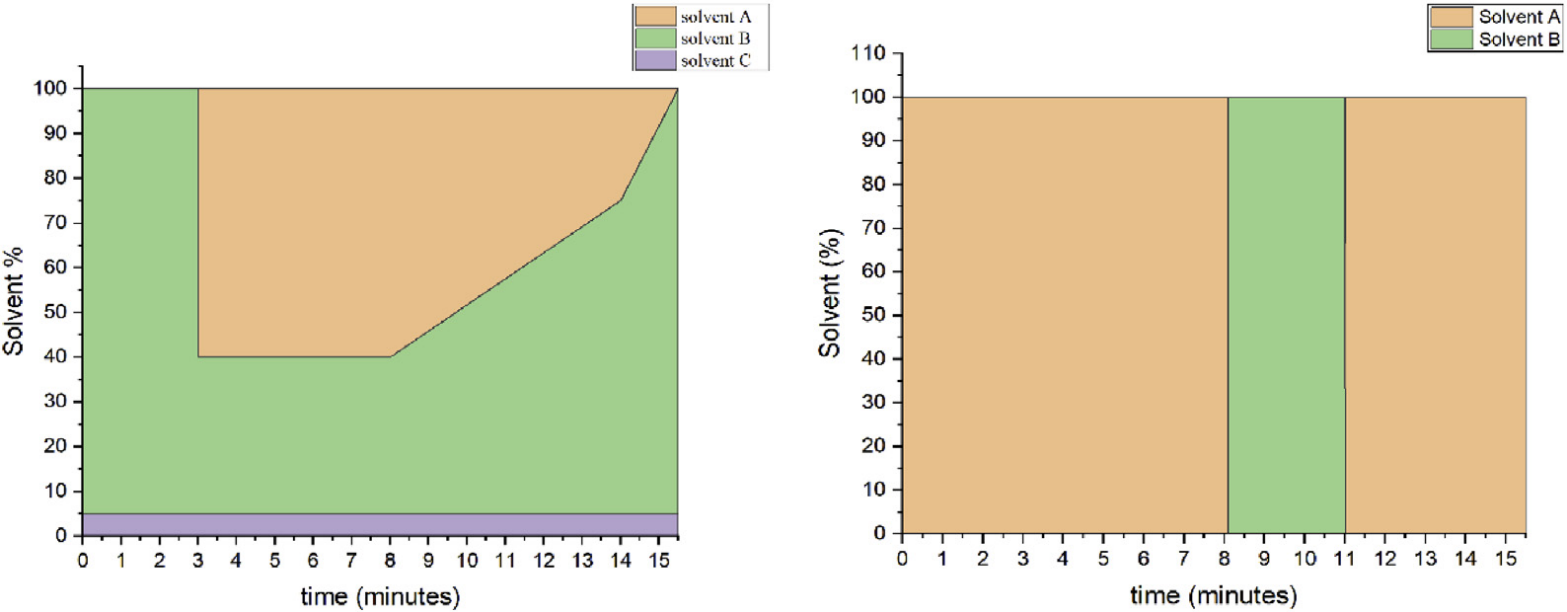
Summary of the gradient program for the optimized online SPE – UHPLC-MS/MS method (left panel: analytical pump; right panel: SPE pump). Analytical pump solvent lines were as follows: solvent A (H_2_O), solvent B (MeOH), solvent C (NH_4_F 1mM in H_2_O). On-line SPE pump solvent lines were as follows: solvent A (HPLC-water with 0.1% HCOOH), solvent B (MeOH).

**Fig. 9:**
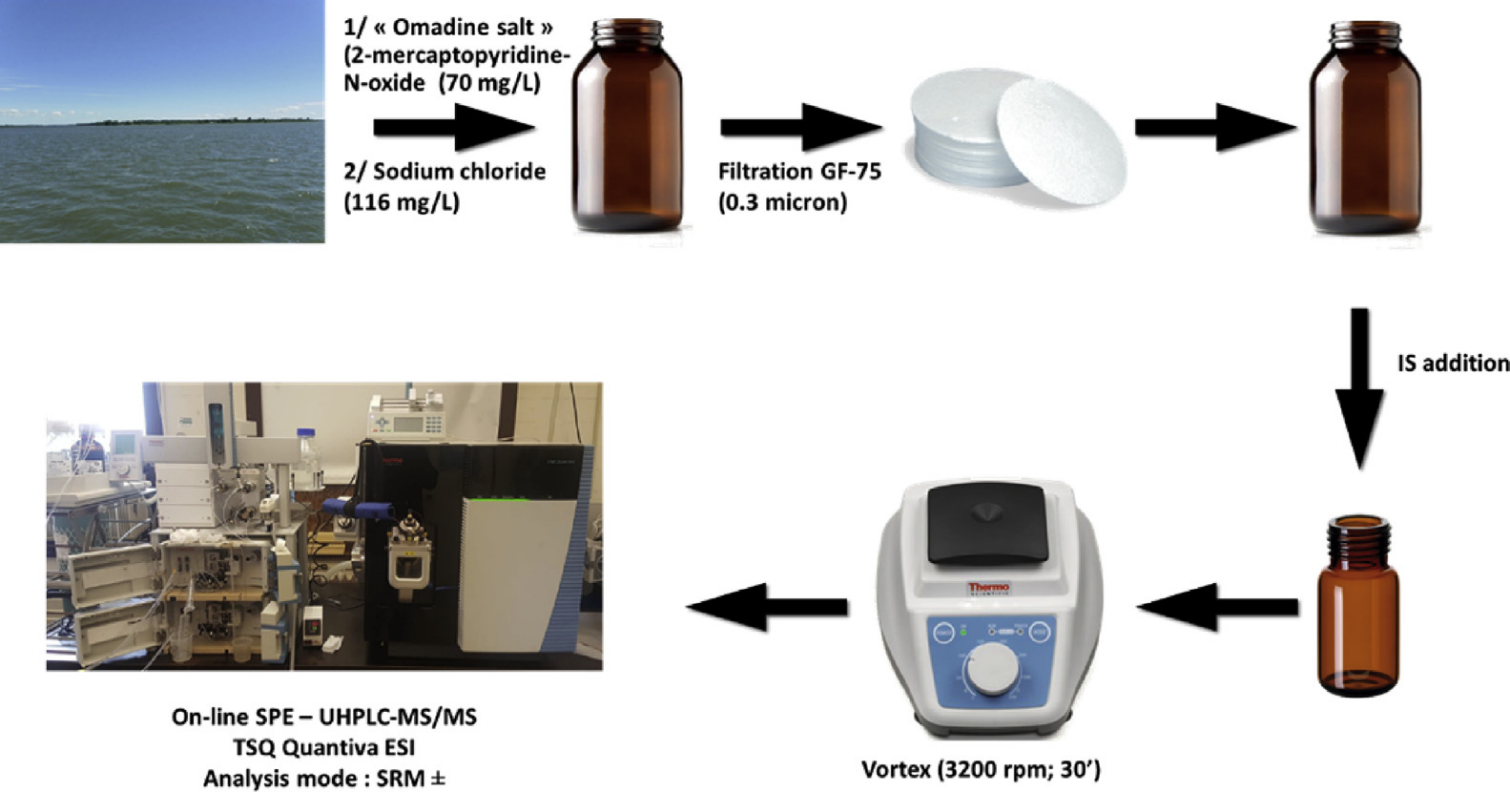
Summary of the sample preparation procedure for surface water.

The following dataset includes 9 figures and 1 table that support the method optimization for the ultra-trace analysis of EPA-priority endocrine disruptors (hormones and bisphenol A). Mass spectrometry optimization is supported by 5 figure elements and one table. Fig. 1, Fig. 2 show the acquisition reports for the optimization of MS/MS parameters (sheath gas, auxiliary gas, precursor ion signal, collision energy, etc.). Fig. 3 shows the signal intensity of targeted compounds when using different sources. Fig. 4 presents the absolute area of each compound analysed under different mass spectrometry conditions in separate acquisition mode *vs.* combined positive/negative fast polarity-switching mode. Fig. 5 highlights the normalized response of the targeted endocrine disruptor compounds related with the concentration of ammonium fluoride (NH_4_F). Table 1 provides the experimental details on compound-dependent MS/MS acquisition conditions. Chromatographic optimization is supported by 3 data files. Fig. 6 provides the UHPLC-MS/MS chromatographic peaks in point by point view while Fig. 7 shows chromatograms illustrating the separation of α-estradiol and β-estradiol. A summary of the chromatographic gradient program is presented in Fig. 8. The overall sample preparation is summarized in Fig. 9.

**Table 1:** Summary of compound-dependent mass spectrometry parameters, including ionization mode, precursor and fragment ions, RF lens, collision energy, and ratio of quantification to confirmation ions (QT/CT) since two MS/MS transitions were followed. The targeted compounds were acquired within a single UHPLC-MS/MS injection, using a heated-ESI source operated in fast-polarity switching.

Compounds	Ionization	Precursor ion	Fragment ion	Transition type	RF lens	Collision energy	Ratio
		(m/z)	(m/z)		(V)	(V)	(QT/CT)
Estriol (E3)	–	287	145	CT	105	43	1.63
			171	QT	105	39	
Bisphenol A (BPA)	–	227	133	CT	66	28	3.03
			212	QT	66	19	
Equilenin (EQUIL)	–	265	221	QT	82	36	2.87
			222	CT	82	29	
Equilin (EQUI)	–	267	143	CT	75	35	4.78
			265	QT	75	25	
Androstenedione (ANDRO)	+	287	97	QT	52	24	1.34
			109	CT	52	26	
β-estradiol (β-E2)	–	271	145	CT	88	42	1.68
			183	QT	88	42	
Estrone (E1)	–	269	145	QT	97	41	1.61
			159	CT	97	39	
Ethinylestradiol (EE2)	–	295	145	QT	97	43	1.55
			159	CT	97	37	
Norethindrone (NOR)	+	299	91	QT	56	44	1.68
			128	CT	56	53	
α-estradiol (α-E2)	–	271	145	QT	95	44	1.68
			183	CT	95	39	
Testosterone (TESTO)	+	289	97	CT	54	24	1.09
			109	QT	54	27	
Levonorgestrel (LEVO)	+	313	91	QT	56	45	1.7
			128	CT	56	59	
Medroxyprogesterone (MEDRO)	+	345	97	CT	58	28	3.59
			123	QT	58	27	
Progesterone (PROG)	+	315	97	CT	55	24	1
			109	QT	55	27

## 2 Experimental design, materials and methods

### 2.1 Mass spectrometry optimization

The tested mass spectrometry conditions are also described in our related research [1]. The herein data presents complementary information on the optimization steps for sheath gas, auxiliary gas, sweep gas, and the spray voltage (Fig. 1). Optimization of the product ion signal and the precursor ion signal was conducted, as was the optimization of collision energy (Fig. 2). The experimental design for the investigation of ionization source type and mobile phase conditions was established based on literature precedent [2], [3], [4]. Fig. 3 presents the variation of signal intensity depending on the selected source, atmospheric pressure chemical ionization (APCI) or heated electrospray ionization (heated-ESI), in combination with different mobile phases: H_2_O/MeOH/NH_4_F (20 mM) or H_2_O+0.1% HCOOH/MeOH. The comparison of signal intensity obtained with polarity-switching ionization mode *vs.* separate mode acquisition is supported by Fig. 4. As discussed in our related study [1], the concentration of ammonium fluoride (NH_4_F) in the LC mobile phase was optimized. The concentration of NH_4_F was investigated at 6 levels (0–20 mM; concentration in line C), and normalized compound-dependent responses are illustrated in Fig. 5. Mass spectrometry parameters with the optimized method are provided in Table 1, which also includes details on relative responses of quantification and confirmation MS/MS transitions (QT/CT ratios).

### 2.2 Chromatographic performance

In accordance with U.S. EPA criteria we verified that each UHPLC-MS/MS chromatographic peak had a minimum of 10 data points (Fig. 6) [3]. The separation of α-estradiol and β-estradiol isomers is illustrated in Fig. 7. A summary of the gradient program used in the optimized on-line SPE – UHPLC-MS/MS method is provided in Fig. 8.

### 2.3 Sample preparation and analysis

The overall procedure for sample preparation is presented in Fig. 9. The sampling steps were previously described [5]. Briefly, at each sampling site the sample was collected in a 1L amber glass bottle and amended with 1 mL of NaCl aqueous solution at 116 g L^−1^ and 1 mL of Omadine salt (2-mercaptopyridine-N-oxide sodium salt) aqueous solution at 70 g L^−1^. The samples were then capped, hand-shaken, and stored at 4 °C until arrival at the laboratory. The samples were passed through 0.3 μm glass fiber filters (GFF-75). The samples were then spiked with the isotope-labelled internal standards (IS) mixture (corresponding to an added quantity of 1.25 ng for each IS) and submitted to high-speed agitation (30 seconds, 3200 rpm) using a LP Vortex mixer from Thermo Scientific. The different types of samples, including tap water, surface water, and wastewater [1], were then analyzed as follows.

The samples were submitted to on-line solid phase extraction (SPE) coupled to ultra-high-performance liquid chromatography tandem mass spectrometry (UHPLC-MS/MS) through a polarity-switching ionization source. A total analysis time of 15.5 minutes per sample was achieved.

The sample delivery system comprised a dual switching-column array. In-loop sample injection was performed with an HTC thermopal autosampler (CTC Analytics AG, Zwingen, Switzerland). The column-switching system [6] was composed of two-position six-port and ten-port valves (VICI Valco Instruments Co., Inc., Houston, TX, U.S.A.). The injection volume was set at 10 mL. An Accela 600 quaternary pump (Thermo Fisher, San Jose, CA, U.S.A.) was used to transfer the sample from the loop to the on-line enrichment column. On-line SPE was achieved using two Hypersil Gold aQ C18 columns (20 mm × 2 mm, 12 μm particle size) connected in series. The on-line SPE mobile phases were HPLC-water with 0.1% formic acid (A) and methanol (B). The gradient program (Fig. 8) comprised three sequential steps: i) the on-line SPE loading (at 1500 μL min^−1^) and washing step; ii) the elution of analytes and separation onto the analytical column; and iii) the conditioning of the analytical column and on-line SPE column prior to the following injection. The injection syringe and injector were washed with a 1:1:1 ACN:MeOH:IPA mixture and with HPLC-water containing 0.1% HCOOH prior to the next injection.

An Accela 1250 quaternary pump (Thermo Finnigan, San Jose, CA, U.S.A.) was used for sample elution from the enrichment column and subsequent separation on the analytical column. Analyte separation was performed using a Thermo Hypersil Gold C18 column (100 mm × 2.1 mm, 1.9 μm particle size) from Thermo Fisher Scientific (San Jose, CA, U.S.A.). The analytical column was thermostated at 55 °C and the mobile phases flow rate set at 500 μL·min^−1^. The analytical mobile phases were HPLC-water (A), methanol (B) and HPLC-water with ammonium fluoride at 1mM (C). Details on the applied gradient program are supplied in Fig. 8.

The TSQ Quantiva triple quadrupole mass spectrometer (Thermo Scientific, Waltham, MA, U.S.A.) was coupled to a heated electrospray ionization source (heated-ESI), operated in fast polarity-switching mode. Source parameters under the optimized conditions were as follows: sheath gas (60 arbitrary unit), auxiliary gas (15 arbitrary unit), sweep gas (0 arbitrary unit), ion spray voltage (+3kV or -3kV, polarity-switching), capillary temperature (350 °C), vaporizer temperature (400 °C). The scan time was set at 20 ms. The first and third quadrupole (Q1 and Q3) were set at unit resolution (0.7 Da FWHM). The collision gas pressure in the collision cell (q2) was fixed at 1.5 mTorr. The analyzer was operated in selected reaction monitoring (SRM) mode, and two MS/MS transitions were monitored for each compound [1]. Compound-dependent MS/MS parameters with the retained method are provided in Table 1.
